# Notes and Notices

**Published:** 1890-11

**Authors:** 


### NOTES AND NOTICES.

Grace Hospital, Detroit.—The next regular examinations for position of Assistant to the House Surgeon will be held at the Hospital, on Thursday, November 13th, at 8.30 p. m. The term of service is eighteen months: first six months as Junior Assistant; second six months as Senior Assistant; third six months as House Surgeon.

Applicants must show evidence of graduation from a recognized Homœopathic College.

All applications to be addressed to the President of the Medical Board, The Grace Hospital, Detroit, Mich., and must be presented not later than November 10th, accompanied by certificate of good moral character.

Removals.—Dr. L. H. Lemke has removed from Warrenton, Mo., to Higginsville, Lafayette County, Mo. Dr. H. A. Mumaw has removed from Orrville, Ohio, to Detroit, Michigan. Dr. Herbert Beals from 370 Michigan Street to 160 Franklin Street, Buffalo, New York. Dr. H. W. Andrews from Chillicothe, Illinois, to Spokane Falls, State of Washington. Dr. W. S. Gee has returned from a brief sojourn at Manitou Springs, Colorado, to his office, 5226 Washington Avenue, Chicago. Dr. S. M. Cate from Salem to Harvard, Mass. Dr. Lawrence M. Stanton from 120 West 129th Street to 71 West 88th Street, New York. Dr. Frank Kraft, in consequence of his appointment as Professor of Materia Medica in the Cleveland College, as announced in our October number, page 437, has removed his office from Sylvania, Ohio, to 29 Euclid Avenue, Cleveland, Ohio. Dr. Charles Deady has removed to 59 West 49th Street, New York. He treats diseases of eye and ear exclusively.

### FUN FOR DOCTORS.

Remarkable Generosity—A.—"A more deserving medical man than our friend Richard does not exist. He very frequently accepts no fees from his patients!"

B.—" You don't say so!"

A.—" For he generally settles with the heirs."—*Fliegende Blätter.*

Dr. Limboff—" Miss Clara, I heard you quoting: ' And sat like Patience on a monument.' Now what does that mean? I never saw Patience on a monument."

Miss Clara—" No, doctor, but I fancy you are quite accustomed to seeing a monument on your patients."—*The Jester.*

